# Supplementary material for: Qualitative study of GPs’ views and experiences of population-based preconception expanded carrier screening in the Netherlands: bioethical perspectives
Source: BMJ Open. 2021 Dec 9;11(12):e056869. doi: 10.1136/bmjopen-2021-056869 (PMC8663082; doi:10.1136/bmjopen-2021-056869)
Supplement: Supplementary data [file bmjopen-2021-056869supp001.pdf]

Supplementary file. Semi-structured interview-guide

(Title of article: *A qualitative study of GPs' views and experiences of population-based preconception expanded carrier screening in the Netherlands: Bioethical perspectives*)

Due to the semi-structured format of the interviews the topics and examples of questions are stated.

First impression of the test
1. Could you describe how you got involved in the PCS project? When did you first hear about it?

Implications of the test
2. Can you describe a general case when someone asks for the screening?

3. How do you explain to people what the test involves?

4. Could you describe different aspects of the test?

Experiences with patients
6. How did couples give meaning to a positive result?

7. How did couples give meaning to a negative result?

8. Did you discuss with the patients the different ways in which the results could be interpreted?

How the test could be improved
9. What do you think should be adjusted in how the test is offered now?

10. How do you think the future of the test looks like?

Other questions
Is there anything we haven't discussed that you would like to add?
