# Supplementary material for: Qualitative study of GPs’ views and experiences of population-based preconception expanded carrier screening in the Netherlands: bioethical perspectives
Source: BMJ Open. 2021 Dec 9;11(12):e056869. doi: 10.1136/bmjopen-2021-056869 (PMC8663082; doi:10.1136/bmjopen-2021-056869)
Supplement: Author's manuscript [file bmjopen-2021-056869.draft_revisions.pdf]

BMJ Open is committed to open peer review. As part of this commitment we make the peer review history of every article we publish publicly available.

When an article is published we post the peer reviewers' comments and the authors' responses online. We also post the versions of the paper that were used during peer review. These are the versions that the peer review comments apply to.

The versions of the paper that follow are the versions that were submitted during the peer review process. They are not the versions of record or the final published versions. They should not be cited or distributed as the published version of this manuscript.

BMJ Open is an open access journal and the full, final, typeset and author-corrected version of record of the manuscript is available on our site with no access controls, subscription charges or pay-per-view fees (http://bmjopen.bmj.com).

If you have any questions on BMJ Open's open peer review process please email info.bmjopen@bmj.com

BMJ Open

A qualitative study of GPs' views and experiences of population-based preconception expanded carrier screening in the Netherlands: Bioethical perspectives

Journal:	*BMJ Open*
Manuscript ID	bmjopen-2021-056869
Article Type:	Original research
Date Submitted by the Author:	01-Sep-2021
Complete List of Authors:	Morberg Jämterud, Sofia; Linköping University, Department of Thematic Studies<br>Snoek, Anke; Maastricht University, Department of Health, Ethics and Society<br>van Langen, I.M.; University of Groningen, Department of Genetics<br>Verkerk, Marian; University of Groningen, Department of Internal Medicine, University Medical Center Groningen<br>Zeiler, Kristin; Linköping University, Department of Thematic Studies
Keywords:	GENETICS, MEDICAL ETHICS, QUALITATIVE RESEARCH, REPRODUCTIVE MEDICINE

SCHOLARONE™
Manuscripts

I, the Submitting Author has the right to grant and does grant on behalf of all authors of the Work (as defined in the below author licence), an exclusive licence and/or a non-exclusive licence for contributions from authors who are: i) UK Crown employees; ii) where BMJ has agreed a CC-BY licence shall apply, and/or iii) in accordance with the terms applicable for US Federal Government officers or employees acting as part of their official duties; on a worldwide, perpetual, irrevocable, royalty-free basis to BMJ Publishing Group Ltd ("BMJ") its licensees and where the relevant Journal is co-owned by BMJ to the co-owners of the Journal, to publish the Work in this journal and any other BMJ products and to exploit all rights, as set out in our licence.

The Submitting Author accepts and understands that any supply made under these terms is made by BMJ to the Submitting Author unless you are acting as an employee on behalf of your employer or a postgraduate student of an affiliated institution which is paying any applicable article publishing charge ("APC") for Open Access articles. Where the Submitting Author wishes to make the Work available on an Open Access basis (and intends to pay the relevant APC), the terms of reuse of such Open Access shall be governed by a Creative Commons licence – details of these licences and which Creative Commons licence will apply to this Work are set out in our licence referred to above.

Other than as permitted in any relevant BMJ Author's Self Archiving Policies, I confirm this Work has not been accepted for publication elsewhere, is not being considered for publication elsewhere and does not duplicate material already published. I confirm all authors consent to publication of this Work and authorise the granting of this licence.

For peer review only

Title page

Title: *A qualitative study of GPs' views and experiences of population-based preconception expanded carrier screening in the Netherlands: Bioethical perspectives*

Keywords: Empirical bioethics, general practitioners, genetics, preconception expanded carrier screening, qualitative interview study

Authors: Morberg Jämterud, Sofia[1], PhD; Snoek, Anke[2], PhD; van Langen, Irene[3], MD, PhD; Verkerk, Marian A.[4], PhD; Zeiler, Kristin[1], PhD.

[1] Linköping University, Department of Thematic Studies, Technology and Social Change, Linköping, Sweden.

[2] Maastricht University, Department of Health, Ethics and Society, Maastricht, The Netherlands.

[3] University of Groningen, Department of Genetics, University Medical Center Groningen, Groningen, The Netherlands.

[4] University of Groningen, Department of Internal Medicine, University Medical Center Groningen, Groningen, The Netherlands.

Corresponding author: Sofia Morberg Jämterud, Linköping University, Department of Thematic Studies, SE-581 83 Linköping, Sweden, sofia.morberg.jamterud@liu.se, ORCID: 0000-0003-2998-3971

Wordcount: 3989

For peer review only

Abstract

Objective: Between 2016 and 2017, population-based preconception expanded carrier screening (PECS) was offered to 4295 couples in the northern Netherlands during a pilot study. It was subsequently made possible in mid-2018 for couples to ask to have such a PECS test from specially trained general practitioners (GPs). Research has described GPs as crucial in offering PECS tests, but little is known about the GPs' views on PECS and their experiences of providing this test. This article presents a thematic analysis of the PECS practice from the perspective of GPs and a bioethical discussion of the empirical results.

Design: Empirical bioethics. A thematic analysis was conducted on qualitative semi-structured interviews, and is combined with an ethical/philosophical discussion.

Setting: The Netherlands.

Participants: 7 Dutch GPs in the Netherlands, interviewed in 2019-2020.

Results: Two themes were identified in the thematic analysis: 'Choice and its complexity' and 'PECS as prompting existential concerns'. The empirical bioethics discussion showed that the first theme highlights that several areas co-shape the complexity of choice on PECS, and the need for shared relational autonomous decision-making on these areas within the couple. The second theme highlights that it is not possible to analyze the existential issues raised by PECS solely on the level of the couple or family. A societal level must be included, since these levels affect each other. We refer to this as 'entangled existential genetics'.

Conclusion: The empirical bioethical analysis leads us to present two practical implications. These are: 1) Training of GPs who are to offer PECS should cover shared relational autonomous decision-making within the couple. 2) More attention should be given to existential issues evoked by genetic considerations, also during the education of GPs and in bioethical discussions around PECS.

For peer review only - http://bmjopen.bmj.com/site/about/guidelines.xhtml

Article summary

Strengths and limitations of this study:

- The qualitative design of the study gives an in-depth perspective on GP's views and experiences of the practice of preconception expanded carrier screening.
- Few qualitative studies that include semi-structured interviews with GPs on preconception expanded carrier screening have been published: this article therefore contributes to this area of research.
- Empirical bioethics as a methodological approach analyses with sensitivity the experiences of GPs of preconception expanded carrier screening in combination with a discussion of the ethical complexities and concerns related to the practice.
- The study involved seven semi-structured interviews which can seem like a small number but was deemed sufficient, since saturation was reached.

For peer review only

INTRODUCTION

Preconception expanded carrier screening (PECS) aims to provide prospective parents with knowledge regarding the risk of conceiving a child with a genetic condition,[1] and to enhance their reproductive autonomy.[2-6] Each child from a couple in which both partners are carriers of a mutation for the same autosomal recessive disorder runs a one in four risk of having the condition. The Netherlands has, as the first country in the world, been offering a couple-based PECS test to non-consanguineous couples who have no known genetic condition in the family. It has been offered by general practitioners (GPs), free of charge, as part of a pilot study.[7, 8] Since mid-2018, all couples in the Netherlands can ask to have a test from one of six specially trained GPs. The test is couple-based, meaning that both partners are tested in parallel, and individual test results are not given. The results are presented solely as the genetic risk that a potential future child would run, as the only aim of the test is to provide results that are important for reproduction.[8] This distinguishes it from many practices with individual-based test results.[9, 10] A couple for whom the result is positive can, for example, choose to be become pregnant following preimplantation genetic diagnosis.[11]

Both in the Netherlands and internationally, GPs have been described as crucial in offering population-based PECS.[12] Some studies have shown that the public prefers that GPs offer the test,[13-15] rather than clinical geneticists or midwives,[11] and it is probable that GPs will receive more inquires about PECS as general knowledge of the practice increases.[16] However, little is known about GPs' views and experiences of PECS. Important qualitative research has been conducted on the views and experiences of GPs related to the feasibility of the test, within an implementation study.[8] The current study has another focus: it examines a wider range of views and experiences of PECS. The article presents a thematic analysis of the PECS practice from the perspective of GPs, and a bioethical discussion of the results.

For peer review only - http://bmjopen.bmj.com/site/about/guidelines.xhtml

METHODS

Setting

In the Netherlands, the PECS test was developed by van Langen and colleagues at the University Medical Centre Groningen (UMCG), who carried out the pilot study.[7, 8, 11, 17] They trained the GPs in non-directive pre-test counselling. The test is not covered by insurance for parents with normal risk, and it now costs EUR 950 per couple. This charge covers the cost of a DNA-lab test performed at UMCG. The scope of the test has increased from 50 serious autosomal recessive genetic conditions to the current 70 conditions. Previous carrier screening tests have often targeted fewer genetic conditions, been offered to specific high-risk groups, or been offered as commercial tests outside of the primary care system.[3, 18]

Data collection and analysis

The study comprised seven semi-structured interviews with GPs. Interviewees were recruited through the UMCG and the Northern GPs' Association (AHON) in the Netherlands. A letter describing the project was sent out to GPs in which interested GPs were asked to contact the research team. Inclusion criteria were working as a GP and willing to share their professional views and reflections on the test. Both GPs who offered the screening and those who only referred couples for it were interviewed. Interviews were conducted by the second author, either in person (September 2019-February 2020) or via phone (March-May 2020). The in-depth interviews lasted around one hour, were recorded, transcribed verbatim, and pseudonymized. Which GP belonged to which group will not be stated in the results in order to ensure anonymity. The study examined the GPs' views and/or experiences on the practice of PECS and the interview guide covered: first impression of the test, implications of the test, experiences with patients, and how the test could be improved.

For peer review only

The qualitative semi-structured design allowed interviewees to expand on issues that they saw as important. The interviews were detailed and rich in content. They offered what, in qualitative research, is called “thick” descriptions, which allow us to consider contextual detail as part of the interpretations and analysis of meaning.[19] The aspect of saturation is essential,[20-22] the later interviews did not bring out new themes, but added to themes already present.[23, 24] For this reason, the number of interviewees was deemed sufficient.

Thematic analysis of the data was conducted.[25] AS, SMJ and KZ read all interviews independently of each other, and carried out an initial coding. AS, SMJ and KZ carried out independent coding of the data, independently identified sub-themes based on this coding, and jointly clustered sub-themes into broader patterns of meaning, i.e. themes. NVivo, software designed to analyze qualitative data, was used. This process, guided by the aim of the study, can be described as the researchers engaging with and interrogating the data, back and forth, and developing themes. The SRQR reporting guidelines have been followed.[26]

For peer review only

Ethics approval statement

The interviewees were informed by letter and orally about the project. Participation was voluntary and participants gave written, informed consent prior to the interview. This article is the result of a Dutch-Swedish collaborative project to examine patients’ views and experiences of PECS, and GP’s work experiences and views of PECS. The sub-study with patients was submitted to and approved by the Medical Ethics Review Board of the UMCG, Groningen (Ref. No. 2019/355), which stated that this study on GPs was not subject to the Dutch Medical Research Involving Human Subjects Act. The sub-study with patients was also submitted to and approved by the Swedish Research Ethics Board (Ref. No. 2019–04501), while the sub-study with healthcare personnel was exempt from review, since it focused on their views and experience of their work, in a way that did not address sensitive

For peer review only - http://bmjopen.bmj.com/site/about/guidelines.xhtml

personal data (personal communication with the scientific secretary of the board, 31 October 2017).

Empirical bioethics

The study's methodological framework is empirical bioethics,[27-29] a growing field of research,[30] where qualitative analysis is combined with an ethical or philosophical analysis.[29, 31] Empirical bioethics has proven to be of much practical value: it entails an iterative process between theory and practice, and ensures close attention to concerns and complexities that arise within a certain practice. These concerns are subsequently refined with philosophical and/or ethical discussion. In the present study, we identify themes that include concerns held by the interviewees, engage with the results of the thematic analysis, identify norms and values, contextualize the identified themes against previous relevant analyses, and discuss the empirical findings in relation to previously identified ethical concerns and discussions.

Patient and public involvement

No patients or members of the public were involved in the study.

RESULTS

Choice and its complexity

GPs stated that they valued PECS because it increases reproductive choice. All stated that non-directiveness was an important ethical condition when offering the screening, underlining the importance of providing adequate information so that the couple could make an informed choice: "We don't force our opinion of matters, but discuss the possibilities and then the choice is theirs" (R1). Some GPs were concerned about whether couples understood what the test tests for and pointed to difficulties in obtaining an overview and understanding of the

For peer review only - http://bmjopen.bmj.com/site/about/guidelines.xhtml

conditions that are screened for, particularly since some of the conditions are very rare. They also wondered whether couples understood what a positive test result implies, namely that they would have to make decisions on reproduction and/or their relationship. GPs were concerned about how the difficulty of grasping such implications affected the couples' informed choice. GPs described that giving adequate information is an important part of explaining the various possible results. Couples needed "at least an understanding that, if something came out [they tested positive], there would be consequences" (R7), and that these consequences may affect the couple's relationship. The GPs were also concerned about the effect of a positive test result on couples and their personal identity, stating that when discussing the range of conditions that were tested for it became clear that "it can also be a huge burden, knowing what genes you carry" (R7).

GPs held the view that attention should be given to the views and reflections of potential parents about what may happen after a positive test result, and more broadly on their views and vision of life:

> You shouldn't educate people to frighten them, that's not the point, but you have to be honest. However, you also touch – you also touch a philosophical aspect, a spiritual aspect. How do you feel about life? It's just not the same for everyone. A lot of people have some kind of idea about that. They haven't thought about it very deeply, but they do have an idea. For some people, it [the test] just doesn't fit in with their philosophy of life, you know. For a lot of people, I think. (R7)

GPs described how a complex situation arose when couples had different views on the test and struggled with shared decision-making as a couple. One GP described how one couple could not agree on what to do after they had tested positive, and later found out that the

For peer review only - http://bmjopen.bmj.com/site/about/guidelines.xhtml

couple had split up before they were informed about the test results. Another GP recalled how a couple could not agree on whether to take the test:

> Both partners should agree on taking the test. And to one couple I said: "Maybe you should go home and think about it, and you can always decide whether to take the test or not. And if you want to come again and talk about it, you are welcome". […] If they can't agree, they should not do it. Because then if you test positive, you have a problem. (R5)

This quotation also illustrates the potential complexity of making this choice together, as a couple.

Complexity of choice and shared relational autonomous decision-making: An empirical bioethics discussion

The theme of choice described above resonates with a common motivation and argument for PECS that revolves around reproductive autonomy, underlining that parents should be informed about the risks of giving birth to an affected child. Based on this information, one can make an informed choice.[8] However, the results of the thematic analysis point to four areas that co-shape the complexity of choice in relation to PECS, from the perspective of GPs. These areas are presented separately, but are closely intertwined. **1. Medical aspects**. The couples must understand what is being tested: the medical conditions may be rare and it may be difficult for them to get clear grasp of the what the conditions involve. **2. Moral aspects**. This area concerns the moral values and ideals held by the couple, and how they are expressed in the decision of how to act if the result is positive. The choice of whether to undertake PECS must also be related to the couples' views and visions of life. **3. Social aspects**. The implications that this test may have for the couples' relationship, and how personal choice

For peer review only - http://bmjopen.bmj.com/site/about/guidelines.xhtml

relates to societal structures. **4. Psychological aspects**. The GPs pointed to a concern for the burden that may be experienced in knowing that one is a carrier.

Since the test is couple-based, the fact that the decision is shared between the partners is underlined. As noted, respect for autonomy is often a key concern in PECS.[2-6] However, while previous discussions of autonomy have sometimes been based on an individualistic interpretation of autonomy, many have argued that relational aspects of autonomy must be considered.[32, 33] It is pointed out that this is a process in which an independent, self-sufficient patient who reaches a decision without interaction with others is not consistent with the relational character of many forms of healthcare.[34, 35] Conceptions of relational autonomy show how social relationships are significant in developing and exercising autonomy: our very ability to make choices is shaped in and by the social context in which we live, and this ability is constrained by the same context.[32] This ability is also constrained by our embodiment.[36] Relational aspects of autonomy can be unpacked using the concept of shared relational autonomous decision-making, which in this case will be shared decision-making *between the partners*. This specifically addresses conditions for decision-making being *shared,* and draws attention to that which takes place between partners who need to come to a shared decision about PECS. Shared relational autonomous decision-making may, therefore, require that both partners have the ability and opportunity to reflect on their vision of life as a family: what matters to each of them individually, what matters to the other partner, and what matters to them as a couple. They may also need to have the ability and opportunity to engage in a decision-making process together, and they must reach a shared decision that both find acceptable.[37] Shared relational autonomous decision-making can therefore be a complex process involving several aspects such as medical, moral, social and psychological aspects (See figure 1). A broader discussion of the role of families in healthcare is pertinent to this discussion.[38]

For peer review only - http://bmjopen.bmj.com/site/about/guidelines.xhtml

PECS as prompting existential concerns

The second theme was centred on how PECS prompts, produces or can be seen as a response to existential concerns at the levels of the couple/family and of society. This theme consists of two sub-themes: "prevention of suffering" and "the test within the framework of societal concerns".

Prevention of suffering

This first sub-theme was centred on suffering as an existential issue related to PECS. The GPs reflected on the PECS test as a means to prevent possible physical and emotional suffering on the part of the families and the potential future child. Some emphasized the prevention of suffering as a reason for offering the test. One GP stated: "Then you first have to explain […] what you want to prevent through carrier screening", namely "the prevention of much suffering" (R2). The nature of suffering was multilayered, where prevention of physical suffering was one layer. One GP described what one of the genetic conditions that the test can identify (epidermolysis bullosa) can mean for a child:

> …the blister condition […] exists in various forms […] it's a very short life expectancy. The babies, at the moment you touch them, a blister forms. So that means that those children will acquire a burn on more than ninety per cent of their body surface area in practically no time. […]. (R5)

GPs also described the suffering of the family as a whole, stating that living with a severely ill child is not only emotionally difficult, but also brings relational, financial and work-related concerns that can cause great distress:

> If you've been in my profession for a while, you see the extremely disastrous consequences of a new family losing their child to disease. Parents who often experience the full bedside, give up their job, and subsequently never mentally get back on track. (R1)

For peer review only - http://bmjopen.bmj.com/site/about/guidelines.xhtml

Prevention of suffering was presented as a concern relating to the whole family, and as potentially existentially devastating. This way of describing suffering helped to position PECS as a response from the medical care system to a devastating situation.

The test within the framework of societal concerns

The second sub-theme was centered on the feeling of responsibility to society, the risk of a less inclusive society, and blame as an existential issue related to PECS.

GPs reflected on what the opportunity to take a screening test could mean for parents' feelings of responsibility to society, if/when giving birth to a child with a condition that could have been screened for. One GP stated: "I think the parents just have the responsibility of being able to prevent having a handicapped child. That burden, of course, is primarily borne by society as a whole. […] I think the general public has to have an opinion about that, whether you wish to bear these things as a society" (R1). "Balancing" the costs to society of offering a genetic test against the costs to society of providing care for a child with a severe disease was described:

> New ways are continuously being found to perform genetic tests in a simpler manner, and thus more affordable. And, as it grows more affordable ... there's always a kind of balancing act, between how expensive a test is, and how expensive it is to have such a child. That's a fairly cynical consideration, of course. (R5)

Further, GPs were concerned about the possibility that screening may result in a less inclusive society:

> […] you can test to prevent people from getting ill, or that ill people are born. But, at the same time […] you want a more inclusive society for everyone […]. That you shouldn't judge someone if they don't want to take such a test, despite you offering it. (R5)

For peer review only

Some GPs expressed concerns that parents who give birth to a child with a condition that the test could have identified would be blamed for conceiving without having taken the test:

> To what extent should you tell people that they have a risk and that they can prevent this? Because at a certain moment, the situation will arise that there are people who have a child with an illness and they could have prevented this. And this is accompanied by blame and penitence, right? (R2)

Entangled existential genetics: An empirical bioethical discussion

Values and norms that are central in this theme are the value attributed to preventing suffering, norms pertaining to responsibility for knowledge about genetic risks and about preventive measures after obtaining such knowledge, and concerns with normative shifts in society, i.e. shifts in the understanding of what one should do when considering whether to try to become pregnant. In the following we show that the sub-themes, when brought into dialogue with each other, underline an intricate interplay, an entanglement, in which the existential issues raised by PECS rest on *both* the level of the couple/family level *and* on the level of society, and that these two levels influence each other. They are entangled. The concept of entanglement is commonly used to describe a situation in which parts that at first glance are regarded as being separated from one another are actually inseparable and thoroughly intertwined.[39] Concerns on the two levels (couple/family and society) inform each other – and the former cannot be fully understood without the latter. As one such example, research has shown that couples' choices are shaped by social values and norms, which differ between socio-cultural contexts. This means that choices, even if autonomous, are influenced by the context in which one lives, and this context not only makes certain discussions and choices possible, but also sets limits on them.[40] As another example, the view of PECS as a method to prevent suffering needs to be understood against other concerns

For peer review only

and ideas, such as the idea that to be responsible, an act must consider the socio-economic resources of society. When these views are understood relative to each other, they help explain how PECS choices made by couples can come to be positioned as concerns both on the couple/family level and on the societal level – and these levels are entangled. As yet another example, previous research has examined whether PECS results in, or strengthens, the idea that parents have a responsibility to prevent giving birth to a child with a severe condition or disability.[41] A similar concern was raised in the GP interviews, namely that parents may be perceived as having a responsibility to use screening. The notion of entanglement helps to demonstrate the intertwinement of these layers of concern. If a couple understands genetic responsibility to be a societal norm and value, this understanding can influence their choice. Furthermore, the couple's choice can, in turn, result in shifts in societal norms towards less inclusive societies. An analysis in which existential concerns related to PECS are seen as an entanglement between the couple/familial and societal levels can shed light on the family-society dynamic. For this reason, what needs to be focused on is what here has been labelled 'entangled existential genetics', existential dimensions that are prompted or evoked by medical practices such as PECS, and in which the interests of the couple/family and society are entangled.

DISCUSSION

We have shown in this study that the need to acknowledge the complexity of choice in relation to PECS, pointing to the co-shaping of medical, moral, social and psychological areas of concern, and the importance of facilitating shared relational autonomous decision-making within the couple in counselling on PECS. Further, we have discussed 'entangled existential genetics'. This idea emphasizes that the existential issues raised by PECS rest *both* on the level of the couple/family level *and* on the level of society. These two levels influence and

For peer review only - http://bmjopen.bmj.com/site/about/guidelines.xhtml

inform each other – they are entangled – and the former cannot be fully understood without the latter. Normative shifts in society can have an impact on what couples come to perceive as the choices available to them, and this in turn can have consequences for societal views on PECS.

A strength in our study is the methodological approach of empirical bioethics. It allows us to combine qualitative analysis with an ethical discussion, maintaining a detailed analysis of descriptions of PECS by GPs, a group of clinicians that may play a crucial role in offering PECS,[11-15] and an analysis of ethical complexities. The relatively small sample in the interviews could be seen as a limitation. However, we concluded that saturation had been reached, since later interviews did not bring out any new themes. This study is based on the practice of PECS as developed within a pilot study in the northern parts of the Netherlands. This means that it may be difficult to generalize our conclusions to other contexts, especially since the test has been developed as a couple-based test. Our results, however, show several aspects that may be interesting also in the context of individual-based tests, such as the identity of the areas that co-shape the complexity of choice.

Previous research has discussed the complexity of consent to screening for a broad range of conditions with a variety of implications in relation to PECS.[3] Ethical concerns have been voiced about the difficulty of obtaining an overview of the conditions that are screened for, and what these conditions can mean. It has been questioned whether this difficulty hampers or has other negative effects on the patients' informed decision-making.[42] The GPs interviewed acknowledge such complexity of overview. However, our results point to a broader complexity in relation to choice, not limited to obtaining an overview, but where the co-shaping of different areas adds to the complexity of choice. Handling such complexity may add to the difficulty for GPs when providing counselling on PECS. Previous research has

For peer review only - http://bmjopen.bmj.com/site/about/guidelines.xhtml

For peer review only

pointed to the need for education of physicians and GPs if they are to play the role of counsellors on PECS,[8, 43] in particular for the need for an awareness of the genetic competence of GPs when counselling on PECS.[16] However, previous studies have acknowledged to a lesser extent the *complexity of choice* that needs to be acknowledged in counselling on PECS.

We offer two recommendations for future GP practices and bioethical discussions of PECS:

- Careful training in non-directive genetic counselling of GPs should cover *shared relational autonomous decision-making within the couple*. Couples should reach a decision following a discussion within a healthcare setting, with healthcare professionals who have been tasked with facilitating the decision-making. Since couple-screening specifically focuses on the couple, and because partners may have different views and values that affect the decision of whether to undergo screening, a more specific training in how to promote shared relational autonomous decision-making within the couple is called for.
- Attention should be given to what we term *entangled existential genetics*, i.e. existential dimensions prompted or evoked by PECS in which the two levels of couple/family and society are entangled. In counselling this can mean that existential dimensions concerning the meaning of the testing for self-understanding are addressed, and the counselling could include how to live with a positive result. Furthermore, entangled existential genetics highlights the importance of political and socio-ethical reflections on existential concerns in bioethical discussions on PECS.

For peer review only

Competing interests

The authors declare that there are no conflicts of interest.

Acknowledgements

The authors would like to thank the participating GPs and Northern GPs' Association AHON (Nynke Schouwenaar/Dorina van der Kolk (PA).

Funding

This research is part of the research program 'A Feminist Approach to Medical Screening' (Grant no. 2016-00784), which has received funding from the Swedish Research Council (Vetenskapsrådet). We thank the Swedish Research Council for its funding of the research.

Author contributions

All authors approved the content of the manuscript. AS collected the data, while AS, SMJ and KZ conducted the thematic analysis. SMJ and KZ took primary responsibility for writing the section of the manuscript that deals with the empirical bioethics results, and the discussion. IvL contributed medical expertise and ethical perspectives on ECS practice. MV contributed a family ethics perspective, and SMJ, AS, MV and KZ contributed medical ethics expertise.

Data sharing statement

No additional data are available.

For peer review only

REFERENCES

1. Edwards JG, Feldman G, Goldberg J, et al. Expanded carrier screening in reproductive medicine – points to consider: a joint statement of the American College of Medical Genetics and Genomics, American College of Obstetricians and Gynecologists, National Society of Genetic Counselors, Perinatal Quality Foundation, and Society for Maternal-Fetal Medicine. *Obstet Gynecol* 2015;125:653–62.
2. Delatycki MB, Alkuraya F, Archibald A, et al. International perspectives on the implementation of reproductive carrier screening. *Prenat Diagn* 2020;40:301–10.
3. Kraft SA, Duenas D, Wilfond BS, et al. The evolving landscape of expanded carrier screening: challenges and opportunities. *Genet Med* 2019;21:790–97.
4. Van der Hout S, Dondorp W, De Wert G. The aims of expanded universal carrier screening: autonomy, prevention, and responsible parenthood. *Bioethics* 2019;33:568–76.
5. Holtkamp KC, Vos EM, Rigter T, et al. Stakeholder perspectives on the implementation of genetic carrier screening in a changing landscape. *BMC Health Serv Res* 2017;17:146.
6. Henneman L, Borry P, Chokoshvili D, et al. Responsible implementation of expanded carrier screening. *Eur J Hum Genet* 2016;24:e1–e12.
7. Schuurmans J, Birnie E, Ranchor A, et al. GP-provided couple-based expanded preconception carrier screening in the Dutch general population: who accepts the test-offer and why? *Eur J Hum Genet* 2020;28:182–92.
8. Schuurmans J, Birnie E, van den Heuvel LM, et al. Feasibility of couple-based expanded carrier screening offered by general practitioners. *Eur J Hum Genet* 2019;27:691–700.

For peer review only - http://bmjopen.bmj.com/site/about/guidelines.xhtml

9. Capalbo A, Fabiani M, Caroselli S, et al. Clinical validity and utility of preconception expanded carrier screening for the management of reproductive genetic risk in IVF and general population. *Hum Reprod* 2021;36:2050–61.
10. Chan OYM, Leung TY, Cao Y, et al. Expanded carrier screening using next-generation sequencing of 123 Hong Kong Chinese families: a pilot study. *Hong Kong Med J* Published Online First: 18 February 2021. doi:10.12809/hkmj208486
11. Plantinga M, Birnie E, Abbott KM, et al. Population-based preconception carrier screening: how potential users from the general population view a test for 50 serious diseases. *Eur J Hum Genet* 2016;24:1417–23.
12. Delatycki MB, Laing NG., Moore SJ, et al. Preconception and antenatal carrier screening for genetic conditions: the critical role of general practitioners. *Aust J Gen Pract* 2019;48:106–10.
13. Voorwinden JS, Buitenhuis AH, Birnie E, et al. Expanded carrier screening: what determines intended participation and can this be influenced by message framing and narrative information? *Eur J Hum Genet* 2017;25:793–800.
14. Nijmeijer SCM, Conijn T, Lakeman P, et al. Attitudes of the general population towards preconception expanded carrier screening for autosomal recessive disorders including inborn errors of metabolism. *Mol Genet Metab* 2019;126:14–22.
15. Bonneau V, Nizon M, Latypova X, et al. First French study relative to preconception genetic testing: 1500 general population participants' opinion. *Orphanet J Rare Dis* 2021;16:130.
16. Janssens S, Chokoshvili D, Vears D, et al. Attitudes of european geneticists regarding expanded carrier screening. *J Obstet Gynecol Neonatal Nurs* 2017;46:63–71.

For peer review only - http://bmjopen.bmj.com/site/about/guidelines.xhtml

17. Plantinga M, Birnie E, Schuurmans J, et al. Expanded carrier screening for autosomal recessive conditions in health care: arguments for a couple-based approach and examination of couples' views. *Prenat Diagn* 2019;39:369–78.

18. Chokoshvili D, Vears DF, Borry P. Growing complexity of (expanded) carrier screening: direct-to-consumer, physician-mediated, and clinic-based offers. *Best Pract Res Clin Obstet Gynaecol* 2017;44:57–67.

19. Geertz C. *The interpretation of cultures: selected essays*. 3rd ed. New York: Basic Books, 2017.

20. Guest G, Bunce A, Johnson L. How many interviews are enough? an experiment with data saturation and variability. *Field Methods* 2006;18:59–82.

21. Morse JM, Barrett M, Mayan M, et al. Verification strategies for establishing reliability and validity in qualitative research. *Int J Qual Methods* 2002;13–22.

22. Saunders B, Sim J, Kingstone T, et al. Saturation in qualitative research: exploring its conceptualization and operationalization. *Qual Quant* 2018;52:1893–1907.

23. Fusch PI, Ness LR. Are we there yet? Data saturation in qualitative research. *Qual Rep* 2015;20:1408–16.

24. Given LM. *100 questions (and answers) about qualitative research*. Los Angeles, California: Sage, 2016.

25. Braun V, Clarke V. Using thematic analysis in psychology. *Qual Res Psychol* 2006;3:77–101.

26. O'Brien BC, Harris IB, Beckman TJ, et al. Standards for reporting qualitative research: a synthesis of recommendations. *Acad Med* 2014;89:1245–51.

27. Kon AA. The role of empirical research in bioethics. *Am J Bioeth* 2009;9:59–65.

28. Morberg Jämterud S. Human dignity: a study in medical ethics. PhD thesis. Uppsala University, Sweden, 2016.

For peer review only

29. Zeiler K, De Boer M. The empirical and the philosophical in empirical bioethics: time for a conceptual turn. *AJOB Empir Bioeth* 2020;11:11–3.

30. Ives J, Dunn M, Crabb A, eds. *Empirical bioethics: theoretical and practical perspectives*. Cambridge: Cambridge University Press, 2018.

31. Musschenga AW. Empirical ethics, context-sensitivity, and contextualism. *J Med Philos* 2005;30:467–90.

32. Mackenzie C, Stoljar N, eds. *Relational autonomy – feminist perspectives on autonomy, agency, and the social self*. New York: Oxford University Press, 2000.

33. Verkerk MA. The care perspective and autonomy. *Med Health Care Philos* 2001;4:289–94.

34. Dove ES, Kelly S, Lucivero F, et al. Beyond individualism: is there a place for relational autonomy in clinical practice and research? *Clin Ethics* 2017;12:150–65.

35. Sherwin S. A relational approach to autonomy in healthcare. In: Sherwin S, ed. *The politics of women's health: exploring agency and autonomy*. Philadelphia: Temple University Press 1998:19–47.

36. Käll LF, Zeiler K. Bodily relational autonomy. *J Conscious Stud* 2014;21:100–20.

37. Zeiler K. Shared decision-making, gender and new technologies. *Med Health Care Philos* 2007;10:279–87.

38. Verkerk M, Lindemann H, McLaughlin J, eds. *What about the family?: practices of responsibility in care*. New York, NY: Oxford University Press, 2019.

39. Fitzgerald D, Callard F. Entangling the medical humanities. In: Whitehead A, Woods A, eds. *The Edinburgh companion to the critical medical humanities*. Edinburgh: Edinburgh University Press 2016:35–49.

40. Raz AE. *Community genetics and genetic alliances: eugenics, carrier testing, and networks of risk*. Abingdon, Oxon and New York: Routledge, 2010.

For peer review only - http://bmjopen.bmj.com/site/about/guidelines.xhtml

41. Raz AE, Amano Y, Timmermans S. Coming to terms with the imperfectly normal child: attitudes of Israeli parents of screen-positive infants regarding subsequent prenatal diagnosis. *J Community Genet* 2019;10:41–50.

42. De Wert GM, Dondorp WJ, Knoppers BM. Preconception care and genetic risk: ethical issues. *J Community Genet* 2012;3:221–28.

43. Briggs A, Nouri PK, Galloway M, et al. Expanded carrier screening: a current survey of physician utilization and attitudes. *J Assist Reprod Genet* 2018;35:1631–40.

For peer review only

For peer review only - http://bmjopen.bmj.com/site/about/guidelines.xhtml

Figure legends

Figure 1. The empirical bioethics discussion of the theme “Choice and its complexity” showed that several areas – medical, psychological, moral, social – co-shape the complexity of choice on PECS. All these areas need to be reflected on, not only by each individual but also by the couple, together. Hence the couple need to engage in a shared relational autonomous decision-making on these areas.

For peer review only

For peer review only - http://bmjopen.bmj.com/site/about/guidelines.xhtml

Figure 1. Result of analysis - Choice and its complexity and shared relational autonomous decision-making on PECS

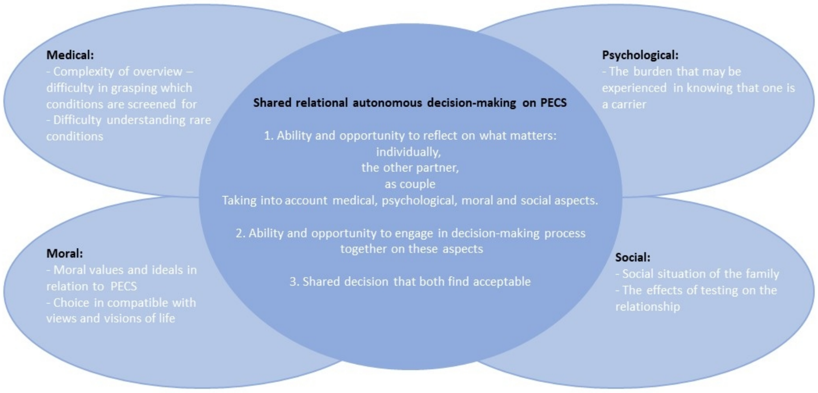

338x190mm (96 x 96 DPI)

Reporting checklist for qualitative study.

Based on the SRQR guidelines.

Instructions to authors

Complete this checklist by entering the page numbers from your manuscript where readers will find each of the items listed below.

Your article may not currently address all the items on the checklist. Please modify your text to include the missing information. If you are certain that an item does not apply, please write "n/a" and provide a short explanation.

Upload your completed checklist as an extra file when you submit to a journal.

In your methods section, say that you used the SRQRreporting guidelines, and cite them as:

O'Brien BC, Harris IB, Beckman TJ, Reed DA, Cook DA. Standards for reporting qualitative research: a synthesis of recommendations. Acad Med. 2014;89(9):1245-1251.

		Reporting Item	Page Number
**Title**			
	#1	Concise description of the nature and topic of the study identifying the study as qualitative or indicating the approach (e.g. ethnography, grounded theory) or data collection methods (e.g. interview, focus group) is recommended	1
**Abstract**			

For peer review only

	#2	Summary of the key elements of the study using the abstract format of the intended publication; typically includes background, purpose, methods, results and conclusions	2
**Introduction**			
Problem formulation	#3	Description and signifcance of the problem / phenomenon studied: review of relevant theory and empirical work; problem statement	4
Purpose or research question	#4	Purpose of the study and specific objectives or questions	4
**Methods**			
Qualitative approach and research paradigm	#5	Qualitative approach (e.g. ethnography, grounded theory, case study, phenomenolgy, narrative research) and guiding theory if appropriate; identifying the research paradigm (e.g. postpositivist, constructivist / interpretivist) is also recommended; rationale. The rationale should briefly discuss the justification for choosing that theory, approach, method or technique rather than other options available; the assumptions and limitations implicit in those choices and how those choices influence study conclusions and transferability. As appropriate	5,6,7

For peer review only

		the rationale for several items might be discussed together.	
Researcher characteristics and reflexivity	#6	Researchers' characteristics that may influence the research, including personal attributes, qualifications / experience, relationship with participants, assumptions and / or presuppositions; potential or actual interaction between researchers' characteristics and the research questions, approach, methods, results and / or transferability	5,6,17
Context	#7	Setting / site and salient contextual factors; rationale	5
Sampling strategy	#8	How and why research participants, documents, or events were selected; criteria for deciding when no further sampling was necessary (e.g. sampling saturation); rationale	5,6
Ethical issues pertaining to human subjects	#9	Documentation of approval by an appropriate ethics review board and participant consent, or explanation for lack thereof; other confidentiality and data security issues	6
Data collection methods	#10	Types of data collected; details of data collection procedures including (as appropriate) start and stop dates of data collection and analysis, iterative process, triangulation of	5,6,7

For peer review only

		sources / methods, and modification of procedures in response to evolving study findings; rationale	
Data collection instruments and technologies	#11	Description of instruments (e.g. interview guides, questionnaires) and devices (e.g. audio recorders) used for data collection; if / how the instruments(s) changed over the course of the study	5
Units of study	#12	Number and relevant characteristics of participants, documents, or events included in the study; level of participation (could be reported in results)	5,6
Data processing	#13	Methods for processing data prior to and during analysis, including transcription, data entry, data management and security, verification of data integrity, data coding, and anonymisation / deidentification of excerpts	5
Data analysis	#14	Process by which inferences, themes, etc. were identified and developed, including the researchers involved in data analysis; usually references a specific paradigm or approach; rationale	6

For peer review only

Techniques to enhance trustworthiness	#15	Techniques to enhance trustworthiness and credibility of data analysis (e.g. member checking, audit trail, triangulation); rationale	6,7
**Results/findings**			
Syntheses and interpretation	#16	Main findings (e.g. interpretations, inferences, and themes); might include development of a theory or model, or integration with prior research or theory	7-14
Links to empirical data	#17	Evidence (e.g. quotes, field notes, text excerpts, photographs) to substantiate analytic findings	7-14
**Discussion**			
Intergration with prior work, implications, transferability and contribution(s) to the field	#18	Short summary of main findings; explanation of how findings and conclusions connect to, support, elaborate on, or challenge conclusions of earlier scholarship; discussion of scope of application / generalizability; identification of unique contributions(s) to scholarship in a discipline or field	14-16 (in empirical bioethics this discussion is also included in the result
Limitations	#19	Trustworthiness and limitations of findings	15
**Other**			
Conflicts of interest	#20	Potential sources of influence of perceived influence on study conduct and conclusions; how these were managed	17

For peer review only

Funding	#21	Sources of funding and other support; role of funders in data collection, interpretation and reporting	17

The SRQR checklist is distributed with permission of Wolters Kluwer © 2014 by the Association of American Medical Colleges. This checklist was completed on 01. July 2020 using https://www.goodreports.org/, a tool made by the EQUATOR Network in collaboration with Penelope.ai

For peer review only

BMJ Open

A qualitative study of GPs' views and experiences of population-based preconception expanded carrier screening in the Netherlands: Bioethical perspectives

Journal:	*BMJ Open*
Manuscript ID	bmjopen-2021-056869.R1
Article Type:	Original research
Date Submitted by the Author:	09-Nov-2021
Complete List of Authors:	Morberg Jämterud, Sofia; Linköping University, Department of Thematic Studies<br>Snoek, Anke; Maastricht University, Department of Health, Ethics and Society<br>van Langen, I.M.; University of Groningen, Department of Genetics<br>Verkerk, Marian; University of Groningen, Department of Internal Medicine, University Medical Center Groningen<br>Zeiler, Kristin; Linköping University, Department of Thematic Studies
**Primary Subject Heading**:	Ethics
Secondary Subject Heading:	Genetics and genomics, Reproductive medicine
Keywords:	GENETICS, MEDICAL ETHICS, QUALITATIVE RESEARCH, REPRODUCTIVE MEDICINE

SCHOLARONE™
Manuscripts

For peer review only - http://bmjopen.bmj.com/site/about/guidelines.xhtml

I, the Submitting Author has the right to grant and does grant on behalf of all authors of the Work (as defined in the below author licence), an exclusive licence and/or a non-exclusive licence for contributions from authors who are: i) UK Crown employees; ii) where BMJ has agreed a CC-BY licence shall apply, and/or iii) in accordance with the terms applicable for US Federal Government officers or employees acting as part of their official duties; on a worldwide, perpetual, irrevocable, royalty-free basis to BMJ Publishing Group Ltd ("BMJ") its licensees and where the relevant Journal is co-owned by BMJ to the co-owners of the Journal, to publish the Work in this journal and any other BMJ products and to exploit all rights, as set out in our licence.

The Submitting Author accepts and understands that any supply made under these terms is made by BMJ to the Submitting Author unless you are acting as an employee on behalf of your employer or a postgraduate student of an affiliated institution which is paying any applicable article publishing charge ("APC") for Open Access articles. Where the Submitting Author wishes to make the Work available on an Open Access basis (and intends to pay the relevant APC), the terms of reuse of such Open Access shall be governed by a Creative Commons licence – details of these licences and which Creative Commons licence will apply to this Work are set out in our licence referred to above.

Other than as permitted in any relevant BMJ Author's Self Archiving Policies, I confirm this Work has not been accepted for publication elsewhere, is not being considered for publication elsewhere and does not duplicate material already published. I confirm all authors consent to publication of this Work and authorise the granting of this licence.

For peer review only

Title page

Title: *A qualitative study of GPs' views and experiences of population-based preconception expanded carrier screening in the Netherlands: Bioethical perspectives*

Keywords: Empirical bioethics, general practitioners, genetics, preconception expanded carrier screening, qualitative interview study

Authors: Morberg Jämterud, Sofia[1], PhD; Snoek, Anke[2], PhD; van Langen, Irene[3], MD, PhD; Verkerk, Marian A.[4], PhD; Zeiler, Kristin[1], PhD.

[1] Linköping University, Department of Thematic Studies, Technology and Social Change, Linköping, Sweden.

[2] Maastricht University, Department of Health, Ethics and Society, Maastricht, The Netherlands.

[3] University of Groningen, Department of Genetics, University Medical Center Groningen, Groningen, The Netherlands.

[4] University of Groningen, Department of Internal Medicine, University Medical Center Groningen, Groningen, The Netherlands.

Corresponding author: Sofia Morberg Jämterud, Linköping University, Department of Thematic Studies, SE-581 83 Linköping, Sweden, sofia.morberg.jamterud@liu.se, ORCID: 0000-0003-2998-3971

Wordcount: 4276

Abstract

Objective: Between 2016 and 2017, a population-based preconception expanded carrier screening (PECS) test was developed in the Netherlands during a pilot study. It was subsequently made possible in mid-2018 for couples to ask to have such a PECS test from

For peer review only

specially trained general practitioners (GPs). Research has described GPs as crucial in offering PECS tests, but little is known about the GPs' views on PECS and their experiences of providing this test. This article presents a thematic analysis of the PECS practice from the perspective of GPs and a bioethical discussion of the empirical results.

Design: Empirical bioethics. A thematic analysis was conducted on qualitative semi-structured interviews, and is combined with an ethical/philosophical discussion.

Setting: The Netherlands.

Participants: 7 Dutch GPs in the Netherlands, interviewed in 2019-2020.

Results: Two themes were identified in the thematic analysis: 'Choice and its complexity' and 'PECS as prompting existential concerns'. The empirical bioethics discussion showed that the first theme highlights that several areas co-shape the complexity of choice on PECS, and the need for shared relational autonomous decision-making on these areas within the couple. The second theme highlights that it is not possible to analyze the existential issues raised by PECS solely on the level of the couple or family. A societal level must be included, since these levels affect each other. We refer to this as 'entangled existential genetics'.

Conclusion: The empirical bioethical analysis leads us to present two practical implications. These are: 1) Training of GPs who are to offer PECS should cover shared relational autonomous decision-making within the couple. 2) More attention should be given to existential issues evoked by genetic considerations, also during the education of GPs and in bioethical discussions around PECS.

Article summary

Strengths and limitations of this study:

- The qualitative design of the study gives an in-depth perspective on GP's views and experiences of the practice of preconception expanded carrier screening.

For peer review only

- Few qualitative studies that include semi-structured interviews with GPs on preconception expanded carrier screening have been published: this article therefore contributes to this area of research.
- Empirical bioethics as a methodological approach analyses with sensitivity the experiences of GPs of preconception expanded carrier screening in combination with a discussion of the ethical complexities and concerns related to the practice.
- The study involved seven semi-structured interviews which can seem like a small number but was deemed sufficient, since saturation was reached.

For peer review only

INTRODUCTION

Preconception expanded carrier screening (PECS) aims to provide prospective parents with knowledge regarding the risk of conceiving a child with a genetic condition,[1] and to enhance their reproductive autonomy.[2-6] Each child from a couple in which both partners are carriers of a mutation for the same autosomal recessive disorder runs a one in four risk of having the condition. The Netherlands has, as the first country in the world, been offering a couple-based PECS test to non-consanguineous couples who have no known genetic

condition in the family. It has been offered preconceptionally by general practitioners (GPs), free of charge, as part of a pilot study and it also included pretest counselling.[7, 8] Since mid-2018, all couples in the Netherlands can ask to have a test from one of six specially trained GPs. The test is couple-based, meaning that both partners are tested in parallel, and individual test results are not given. The results are presented solely as the genetic risk that a potential future child would run (carrier couple or not), as the only aim of the test is to provide results that are important for reproduction.[8] This distinguishes it from many practices with individual-based test results.[9, 10] A couple for whom the result is positive can, for example, choose to be become pregnant following IVF combined with preimplantation genetic diagnosis.[11]

Both in the Netherlands and internationally, GPs have been described as crucial in offering population-based PECS.[12] Some studies have shown that the public prefers that GPs offer the test,[13-15] rather than clinical geneticists or midwives,[11] and it is probable that GPs will receive more inquiries about PECS as general knowledge of the practice increases.[16] However, little is known about GPs' views and experiences of PECS. Previous qualitative research has been conducted on the views and experiences of GPs related to the feasibility of the test, within an implementation study.[8] However, the current study has another focus: it examines a wider range of views and experiences of PECS and does not only focus on feasibility of the test. The aim of this article is to present an empirical bioethics analysis of the PECS practice from the perspective of GPs.

METHODS

Setting

In the Netherlands, the PECS test and (pre-test) counselling was developed by van Langen and colleagues at the University Medical Centre Groningen (UMCG) in 2016, as part of a pilot study.[7, 8, 11, 17] They have trained GPs in non-directive pre-test counselling. The GPs do not perform post-test counselling but refers couples to clinical geneticists. The test is not

For peer review only - http://bmjopen.bmj.com/site/about/guidelines.xhtml

covered by insurance for parents with normal risk, and it costs EUR 950 per couple. This charge covers the cost of a DNA-lab test performed at UMCG. The test currently includes 70 serious early onset autosomal recessive genetic conditions. Previous carrier screening tests have often targeted fewer genetic conditions, been offered to specific high-risk groups, or been offered as commercial tests outside of the primary care system.[3, 18] Internationally, much genetic carrier screening is offered in early pregnancy.[19-21] However, this is not the case in this study since the focus is only on a test that is taken before conception.

Data collection and analysis

The study comprised seven semi-structured interviews with GPs. Interviewees were recruited through the UMCG and the Northern GPs' Association (AHON) in the Netherlands. A letter describing the project was sent out to GPs in which interested GPs were asked to contact the research team. Inclusion criteria were working as a GP and willing to share their professional views and reflections on the test. Both GPs who offered the screening and those who only referred couples for it were interviewed. The majority of GPs were situated in rural areas. Interviews were conducted by the second author, either in person (September 2019-February 2020) or via phone (March-May 2020). The in-depth interviews lasted around one hour, were

For peer review only

recorded, transcribed verbatim, and pseudonymized. Which GP belonged to which group will not be stated in the results in order to ensure anonymity. The study examined the GPs' views and/or experiences on the practice of PECS and the interview guide covered: first impression of the test, implications of the test, experiences with patients, and how the test could be improved (supplementary file).

The qualitative semi-structured design allowed interviewees to expand on issues that they saw as important. The interviews were detailed and rich in content. They offered what, in qualitative research, is called "thick" descriptions, which allow us to consider contextual detail as part of the interpretations and analysis of meaning.[22] The aspect of saturation is essential,[23-25] the later interviews did not bring out new themes, but added to themes already present.[26, 27] For this reason, the number of interviewees was deemed sufficient.

Thematic analysis of the data was conducted.[28] AS, SMJ and KZ read all interviews independently of each other, and carried out an initial coding. AS, SMJ and KZ carried out independent coding of the data, independently identified sub-themes based on this coding, and jointly clustered sub-themes into broader patterns of meaning, i.e. themes. NVivo, software designed to analyze qualitative data, was used. This process, guided by the aim of the study, can be described as the researchers engaging with and interrogating the data, back and forth, and developing themes. The SRQR reporting guidelines have been followed.[29]

Ethics approval statement

The interviewees were informed by letter and orally about the project. Participation was voluntary and participants gave written, informed consent prior to the interview. This article is the result of a Dutch-Swedish collaboration to examine patients' views and experiences of PECS, and GP's work experiences and views of PECS. The sub-study with patients was submitted to and approved by the Medical Ethics Review Board of the UMCG, Groningen

For peer review only

(Ref. No. 2019/355), which stated that this study on GPs was not subject to the Dutch Medical Research Involving Human Subjects Act. The sub-study with patients was also submitted to and approved by the Swedish Research Ethics Board (Ref. No. 2019–04501), while the sub-study with healthcare personnel was exempt from review, since it focused on their views and experience of their work, in a way that did not address sensitive personal data (personal communication with the scientific secretary of the board, 31 October 2017).

Empirical bioethics

The study's methodological framework is empirical bioethics,[30-32] a growing field of research.[33] Empirical bioethics is a heterogeneous field that combines empirical research – commonly qualitative empirical research – with an ethical or philosophical analysis.[32, 34] Just as other qualitative research methods, it involves a detailed analysis of descriptions and views given by interviewees on a particular subject, and a focus on complexities. However, the particular value of empirical bioethics rests with the way the qualitative analysis is combined with for example conceptual analysis and philosophical and ethical discussion.[30, 32, 34] The combination of qualitative analyses with philosophical or ethical analyses has proven to be of much value: it can refine an ethical discussion within a medical practice through its close attention to concerns that arise within this practice, without losing sight of the specific context, while ensuring that theoretical philosophical and/or ethical discussions contribute to concerns within the concrete medical practice. In this way, such combined analyses can contribute to the improvement of care. In the present study, we identify themes that include concerns held by the interviewees, engage with the results of the thematic analysis, identify norms and values, contextualize the identified themes against previous

For peer review only - http://bmjopen.bmj.com/site/about/guidelines.xhtml

relevant analyses, and discuss the empirical findings in relation to previously identified ethical concerns and discussions (here called an empirical bioethical discussion).

Patient and public involvement

No patients or members of the public were involved in the study.

RESULTS

The first theme identified in the thematic analysis of interviews with GPs on PECS is “choice and its complexity”. After presenting the thematic analysis we offer an empirical bioethics discussion and argue that it highlights the need for facilitating shared relational autonomous decision-making within the couple. The second theme is “PECS as prompting existential concerns”, which includes two sub-themes: “prevention of suffering” and “the test within the framework of societal concerns”. We also discuss this theme in the context of bioethics and argue that it should preferably be understood in terms of an entangled existential genetics that brings out ethically pertinent aspects of the practice of PECS.

Choice and its complexity

GPs stated that they valued PECS because it increases reproductive choice. All stated that non-directiveness was an important ethical condition when offering the screening, underlining the importance of providing adequate information so that the couple could make an informed choice: “We don't force our opinion of matters, but discuss the possibilities and then the choice is theirs” (R1). Some GPs were concerned about whether couples understood what the test tests for and pointed to difficulties in obtaining an overview and understanding of the conditions that are screened for, particularly since some of the conditions are very rare. They also wondered whether couples understood what a positive test result implies, namely that

For peer review only - http://bmjopen.bmj.com/site/about/guidelines.xhtml

they might have to make decisions on reproduction and/or their relationship. GPs were concerned about how the difficulty of grasping such implications affected the couples' informed choice. GPs described that giving adequate information is an important part of explaining the various possible results. Couples needed "at least an understanding that, if something came out [they tested positive], there would be consequences" (R7), and that these consequences may affect the couple's relationship. The GPs were also concerned about the effect of a positive test result on couples and their personal identity, stating that when discussing the range of conditions that were tested for it became clear that "it can also be a huge burden, knowing what genes you carry" (R7).

GPs held the view that attention should be given to the views and reflections of potential parents about what may happen after a positive test result, and more broadly on their views and vision of life:

> You shouldn't educate people to frighten them, that's not the point, but you have to be honest. However, you also touch – you also touch a philosophical aspect, a spiritual aspect. How do you feel about life? It's just not the same for everyone. A lot of people have some kind of idea about that. They haven't thought about it very deeply, but they do have an idea. For some people, it [the test] just doesn't fit in with their philosophy of life, you know. For a lot of people, I think. (R7)

GPs described how a complex situation arose when couples had different views on the test and struggled with shared decision-making as a couple. One GP described how one couple could not agree on what to do after they had tested positive, and later found out that the couple had split up before they were informed about the test results. Another GP recalled how a couple could not agree on whether to take the test:

For peer review only - http://bmjopen.bmj.com/site/about/guidelines.xhtml

> Both partners should agree on taking the test. And to one couple I said: “Maybe you should go home and think about it, and you can always decide whether to take the test or not. And if you want to come again and talk about it, you are welcome”. […] If they can’t agree, they should not do it. Because then if you test positive, you have a problem. (R5)

This quotation also illustrates the potential complexity of making this choice together, as a couple.

Complexity of choice and shared relational autonomous decision-making: An empirical bioethics discussion

The theme of choice described above resonates with a common motivation and argument for PECS that revolves around reproductive autonomy, underlining that parents should be informed about the risks of giving birth to an affected child. Based on this information, one can make an informed choice.[8] However, the results of the thematic analysis point to four areas that co-shape the complexity of choice in relation to PECS, from the perspective of GPs. These areas are presented separately, but are closely intertwined. **1. Medical aspects**. The couples must understand what is being tested: the medical conditions may be rare and it may be difficult for them to get clear grasp of the what the conditions involve. **2. Moral aspects**. This area concerns the moral values and ideals held by the couple, and how they are expressed in the decision of how to act if the result is positive. The choice of whether to undertake PECS must also be related to the couples’ views and visions of life. **3. Social aspects**. The implications that this test may have for the couples’ relationship, and how personal choice relates to societal structures. **4. Psychological aspects**. The GPs pointed to a concern for the burden that may be experienced in knowing that one is a carrier.

For peer review only - http://bmjopen.bmj.com/site/about/guidelines.xhtml

Since the test is couple-based, the fact that the decision is shared between the partners is underlined. As noted, respect for autonomy is often a key concern in PECS.[2-6] However, while previous discussions of autonomy have sometimes been based on an individualistic interpretation of autonomy, many have argued that relational aspects of autonomy must be considered.[35, 36] It is pointed out that this is a process in which an independent, self-sufficient patient who reaches a decision without interaction with others is not consistent with the relational character of many forms of healthcare.[37, 38] Conceptions of relational autonomy show how social relationships are significant in developing and exercising autonomy: our very ability to make choices is shaped in and by the social context in which we live, and this ability is constrained by the same context.[35] Relational aspects of autonomy can be unpacked using the concept of shared relational autonomous decision-making, which in this case will be shared decision-making *between the partners*. This specifically addresses conditions for decision-making being *shared,* and draws attention to that which takes place between partners who need to come to a shared decision about PECS. Shared relational autonomous decision-making may, therefore, require that both partners have the ability and opportunity to reflect on their vision of life as a family: what matters to each of them individually, what matters to the other partner, and what matters to them as a couple. They may also need to have the ability and opportunity to engage in a decision-making process together, and they must reach a shared decision that both find acceptable.[39] Shared relational autonomous decision-making can therefore be a complex process involving several aspects such as medical, moral, social and psychological aspects (See figure 1). A broader discussion of the role of families in healthcare is pertinent to this discussion.[40]

PECS as prompting existential concerns

The second theme was centred on how PECS prompts, produces or can be seen as a response to existential concerns at the levels of the couple/family and of society. This theme consists of

For peer review only - http://bmjopen.bmj.com/site/about/guidelines.xhtml

two sub-themes: “prevention of suffering” and “the test within the framework of societal concerns”.

Prevention of suffering

This first sub-theme was centred on suffering as an existential issue related to PECS. The GPs reflected on the PECS test as a means to prevent possible physical and emotional suffering on the part of the families and the potential future child. Some emphasized the prevention of suffering as a reason for offering the test. One GP stated: “Then you first have to explain […] what you want to prevent through carrier screening”, namely “the prevention of much suffering” (R2). The nature of suffering was multilayered, where prevention of physical suffering was one layer. One GP described what one of the genetic conditions that the test can identify (epidermolysis bullosa) can mean for a child:

> …the blister condition […] exists in various forms […] it’s a very short life expectancy. The babies, at the moment you touch them, a blister forms. So that means that those children will acquire a burn on more than ninety per cent of their body surface area in practically no time. […]. (R5)

GPs also described the suffering of the family as a whole, stating that living with a severely ill child is not only emotionally difficult, but also brings relational, financial and work-related concerns that can cause great distress:

> If you've been in my profession for a while, you see the extremely disastrous consequences of a new family losing their child to disease. Parents who often experience the full bedside, give up their job, and subsequently never mentally get back on track. (R1)

For peer review only - http://bmjopen.bmj.com/site/about/guidelines.xhtml

Prevention of suffering was presented as a concern relating to the whole family, and as potentially existentially devastating. This way of describing suffering helped to position PECS as a response from the medical care system to a devastating situation.

The test within the framework of societal concerns

The second sub-theme was centered on the feeling of responsibility to society, the risk of a less inclusive society, and blame as an existential issue related to PECS.

GPs reflected on what the opportunity to take a screening test could mean for parents' feelings of responsibility to society, if/when giving birth to a child with a condition that could have been screened for. One GP stated: "I think the parents just have the responsibility of being able to prevent having a handicapped child. That burden, of course, is primarily borne by society as a whole. […] I think the general public has to have an opinion about that, whether you wish to bear these things as a society" (R1). "Balancing" the costs to society of offering a genetic test against the costs to society of providing care for a child with a severe disease was described:

> New ways are continuously being found to perform genetic tests in a simpler manner, and thus more affordable. And, as it grows more affordable ... there's always a kind of balancing act, between how expensive a test is, and how expensive it is to have such a child. That's a fairly cynical consideration, of course. (R5)

Further, GPs were concerned about the possibility that screening may result in a less inclusive society:

> […] you can test to prevent people from getting ill, or that ill people are born. But, at the same time […] you want a more inclusive society for everyone […]. That you shouldn't judge someone if they don't want to take such a test, despite you offering it. (R5)

For peer review only - http://bmjopen.bmj.com/site/about/guidelines.xhtml

Some GPs expressed concerns that parents who give birth to a child with a condition that the test could have identified would be blamed for conceiving without having taken the test:

> To what extent should you tell people that they have a risk and that they can prevent this? Because at a certain moment, the situation will arise that there are people who have a child with an illness and they could have prevented this. And this is accompanied by blame and penitence, right? (R2)

Entangled existential genetics: An empirical bioethical discussion

Values and norms that are central in this theme are the value attributed to preventing suffering, norms pertaining to responsibility for knowledge about genetic risks and about preventive measures after obtaining such knowledge, and concerns with normative shifts in society, i.e. shifts in the understanding of what one should do when considering whether to try to become pregnant. In the following we show that the sub-themes, when brought into dialogue with each other, underline an intricate interplay, an entanglement, in which the existential issues raised by PECS rest on *both* the level of the couple/family level *and* on the level of society, and that these two levels influence each other. They are entangled. The concept of entanglement is commonly used to describe a situation in which parts that at first glance are regarded as being separated from one another are actually inseparable and thoroughly intertwined.[41] Concerns on the two levels (couple/family and society) inform each other – and the former cannot be fully understood without the latter. As one such example, research has shown that couples' choices are shaped by social values and norms, which differ between socio-cultural contexts. This means that choices, even if autonomous, are influenced by the context in which one lives, and this context not only makes certain discussions and choices possible, but also sets limits on them.[42] As another example, the view of PECS as a method to prevent suffering needs to be understood against other concerns

For peer review only - http://bmjopen.bmj.com/site/about/guidelines.xhtml

and ideas, such as the idea that to be responsible, an act must consider societal concerns.. When these views are understood relative to each other, they help explain how PECS choices made by couples can come to be positioned as concerns both on the couple/family level and on the societal level – and these levels are entangled. As yet another example, previous research has examined whether PECS results in, or strengthens, the idea that parents have a responsibility to prevent giving birth to a child with a severe condition or disability.[43] A similar concern was raised in the GP interviews, namely that parents may be perceived as having a responsibility to use screening. The notion of entanglement helps to demonstrate the intertwinement of these layers of concern. If a couple understands genetic responsibility to be a societal norm and value, this understanding can influence their choice. Furthermore, couples' choices can, in turn, result in shifts in societal norms towards less inclusive societies. An analysis in which existential concerns related to PECS are seen as an entanglement between the couple/familial and societal levels can shed light on the family-society dynamic. For this reason, what needs to be focused on is what here has been labelled 'entangled existential genetics', existential dimensions that are prompted or evoked by medical practices such as PECS, and in which the interests of the couple/family and society are entangled.

DISCUSSION

We have shown in this study that the need to acknowledge the complexity of choice in relation to PECS, pointing to the co-shaping of medical, moral, social and psychological areas of concern, and the importance of facilitating shared relational autonomous decision-making within the couple in counselling on PECS. Further, we have discussed 'entangled existential genetics'. This idea emphasizes that the existential issues raised by PECS rest *both* on the level of the couple/family level *and* on the level of society. These two levels influence and inform each other – they are entangled – and the former cannot be fully understood without

For peer review only - http://bmjopen.bmj.com/site/about/guidelines.xhtml

the latter. Normative shifts in society can have an impact on what couples come to perceive as the choices available to them, and this in turn can have consequences for societal views on PECS.

A strength in our study is the methodological approach of empirical bioethics. It allows us to combine qualitative analysis with an ethical discussion, maintaining a detailed analysis of descriptions of PECS by GPs, a group of clinicians that may play a crucial role in offering PECS,[8, 12-15] and an analysis of ethical complexities. The in-depth nature of the interviews is also a strength of this study even though the relatively small sample in the interviews could be seen as a limitation. However, we concluded that saturation had been reached, since later interviews did not bring out any new themes. This study is based on the practice of PECS as developed within a pilot study in the northern parts of the Netherlands. This means that it may be difficult to generalize our conclusions to other contexts, especially since the test has been developed as a couple-based test. Our results, however, show several aspects that may be interesting also in the context of individual-based tests, such as the identity of the areas that co-shape the complexity of choice.

Previous research has discussed the complexity of consent to screening for a broad range of conditions with a variety of implications in relation to PECS.[3] Ethical concerns have been voiced about the difficulty of obtaining an overview of the conditions that are screened for, and what these conditions can mean. It has been questioned whether this difficulty hampers or has other negative effects on the patients' informed decision-making.[44] The GPs interviewed acknowledge such complexity of overview. However, our results point to a broader complexity in relation to choice, not limited to obtaining an overview, but where the co-shaping of different areas adds to the complexity of choice. Handling such complexity may add to the difficulty for GPs when providing counselling on PECS. Previous research has

For peer review only - http://bmjopen.bmj.com/site/about/guidelines.xhtml

pointed to the need for education of physicians and GPs if they are to play the role of counsellors on PECS,[8, 45] in particular for the need for an awareness of the genetic competence of GPs when counselling on PECS.[16] However, previous studies have acknowledged to a lesser extent the *complexity of choice* that needs to be acknowledged in counselling on PECS.

We offer two recommendations for future GP practices and bioethical discussions of PECS:

- Careful training in non-directive genetic counselling of GPs should cover *shared relational autonomous decision-making within the couple*. Couples should reach a decision following a discussion within a healthcare setting, with healthcare professionals who have been tasked with facilitating the decision-making. Since couple-screening specifically focuses on the couple, and because partners may have different views and values that affect the decision of whether to undergo screening, a more specific training in how to promote shared relational autonomous decision-making within the couple is called for.
- Attention should be given to what we term *entangled existential genetics*, i.e. existential dimensions prompted or evoked by PECS in which the two levels of couple/family and society are entangled. In counselling this can mean that existential dimensions concerning the meaning of the testing for self-understanding are addressed, and the counselling could include how to live with a positive result. Furthermore, entangled existential genetics highlights the importance of political and socio-ethical reflections on existential concerns in bioethical discussions on PECS.

For peer review only

For peer review only - http://bmjopen.bmj.com/site/about/guidelines.xhtml

Competing interests

The authors declare that there are no conflicts of interest.

Acknowledgements

The authors would like to thank the participating GPs and Northern GPs' Association AHON (Nynke Schouwenaar/Dorina van der Kolk (PA).

Funding

This research is part of the research program 'A Feminist Approach to Medical Screening' (Grant no. 2016-00784), which has received funding from the Swedish Research Council (Vetenskapsrådet). We thank the Swedish Research Council for its funding of the research.

Author contributions

All authors approved the content of the manuscript. AS collected the data, while AS, SMJ and KZ conducted the thematic analysis. SMJ and KZ took primary responsibility for writing the section of the manuscript that deals with the empirical bioethics results, and the discussion. IvL contributed medical expertise and ethical perspectives on ECS practice. MV contributed a family ethics perspective, and SMJ, AS, MV and KZ contributed medical ethics expertise.

Data sharing statement

No additional data are available.

For peer review only - http://bmjopen.bmj.com/site/about/guidelines.xhtml

REFERENCES

1. Edwards JG, Feldman G, Goldberg J, et al. Expanded carrier screening in reproductive medicine – points to consider: a joint statement of the American College of Medical Genetics and Genomics, American College of Obstetricians and Gynecologists, National Society of Genetic Counselors, Perinatal Quality Foundation, and Society for Maternal-Fetal Medicine. *Obstet Gynecol* 2015;125:653–62.
2. Delatycki MB, Alkuraya F, Archibald A, et al. International perspectives on the implementation of reproductive carrier screening. *Prenat Diagn* 2020;40:301–10.
3. Kraft SA, Duenas D, Wilfond BS, et al. The evolving landscape of expanded carrier screening: challenges and opportunities. *Genet Med* 2019;21:790–97.
4. Van der Hout S, Dondorp W, De Wert G. The aims of expanded universal carrier screening: autonomy, prevention, and responsible parenthood. *Bioethics* 2019;33:568–76.
5. Holtkamp KC, Vos EM, Rigter T, et al. Stakeholder perspectives on the implementation of genetic carrier screening in a changing landscape. *BMC Health Serv Res* 2017;17:146.
6. Henneman L, Borry P, Chokoshvili D, et al. Responsible implementation of expanded carrier screening. *Eur J Hum Genet* 2016;24:e1–e12.
7. Schuurmans J, Birnie E, Ranchor A, et al. GP-provided couple-based expanded preconception carrier screening in the Dutch general population: who accepts the test-offer and why? *Eur J Hum Genet* 2020;28:182–92.
8. Schuurmans J, Birnie E, van den Heuvel LM, et al. Feasibility of couple-based expanded carrier screening offered by general practitioners. *Eur J Hum Genet* 2019;27:691–700.

For peer review only - http://bmjopen.bmj.com/site/about/guidelines.xhtml

9. Capalbo A, Fabiani M, Caroselli S, et al. Clinical validity and utility of preconception expanded carrier screening for the management of reproductive genetic risk in IVF and general population. *Hum Reprod* 2021;36:2050–61.
10. Chan OYM, Leung TY, Cao Y, et al. Expanded carrier screening using next-generation sequencing of 123 Hong Kong Chinese families: a pilot study. *Hong Kong Med J* 2021;27:177-183.
11. Plantinga M, Birnie E, Abbott KM, et al. Population-based preconception carrier screening: how potential users from the general population view a test for 50 serious diseases. *Eur J Hum Genet* 2016;24:1417–23.
12. Delatycki MB, Laing NG., Moore SJ, et al. Preconception and antenatal carrier screening for genetic conditions: the critical role of general practitioners. *Aust J Gen Pract* 2019;48:106–10.
13. Voorwinden JS, Buitenhuis AH, Birnie E, et al. Expanded carrier screening: what determines intended participation and can this be influenced by message framing and narrative information? *Eur J Hum Genet* 2017;25:793–800.
14. Nijmeijer SCM, Conijn T, Lakeman P, et al. Attitudes of the general population towards preconception expanded carrier screening for autosomal recessive disorders including inborn errors of metabolism. *Mol Genet Metab* 2019;126:14–22.
15. Bonneau V, Nizon M, Latypova X, et al. First French study relative to preconception genetic testing: 1500 general population participants' opinion. *Orphanet J Rare Dis* 2021;16:130.
16. Janssens S, Chokoshvili D, Vears D, et al. Attitudes of European geneticists regarding expanded carrier screening. *J Obstet Gynecol Neonatal Nurs* 2017;46:63–71.

For peer review only

For peer review only - http://bmjopen.bmj.com/site/about/guidelines.xhtml

17. Plantinga M, Birnie E, Schuurmans J, et al. Expanded carrier screening for autosomal recessive conditions in health care: arguments for a couple-based approach and examination of couples' views. *Prenat Diagn* 2019;39:369–78.

18. Chokoshvili D, Vears DF, Borry P. Growing complexity of (expanded) carrier screening: direct-to-consumer, physician-mediated, and clinic-based offers. *Best Pract Res Clin Obstet Gynaecol* 2017;44:57–67.

19. Gregg AR, Aarabi M, Klugman S, et al. Screening for autosomal recessive and X-linked conditions during pregnancy and preconception: a practice resource of the American College of Medical Genetics and Genomics (ACMG). *Genet Med* 2021; 23:1793–1806.

20. Kirk EP, Ong R, Boggs K, et al. Gene selection for the Australian reproductive genetic carrier screening project ("Mackenzie's Mission"). *Eur J Hum Genet* 2021;29:79–87.

21. Dive L, Newson A. Ethics of reproductive genetic carrier screening: from the clinic to the population. *Public Health Ethics* 2021;14:202–17.

22. Geertz C. *The interpretation of cultures: selected essays*. 3rd ed. New York: Basic Books, 2017.

23. Guest G, Bunce A, Johnson L. How many interviews are enough? An experiment with data saturation and variability. *Field Methods* 2006;18:59–82.

24. Morse JM, Barrett M, Mayan M, et al. Verification strategies for establishing reliability and validity in qualitative research. *Int J Qual Methods* 2002;1:13–22.

25. Saunders B, Sim J, Kingstone T, et al. Saturation in qualitative research: exploring its conceptualization and operationalization. *Qual Quant* 2018;52:1893–1907.

26. Fusch PI, Ness LR. Are we there yet? Data saturation in qualitative research. *Qual Rep* 2015;20:1408–16.

For peer review only - http://bmjopen.bmj.com/site/about/guidelines.xhtml

27. Given LM. *100 questions (and answers) about qualitative research*. Los Angeles, California: Sage, 2016.

28. Braun V, Clarke V. Using thematic analysis in psychology. *Qual Res Psychol* 2006;3:77–101.

29. O'Brien BC, Harris IB, Beckman TJ, et al. Standards for reporting qualitative research: a synthesis of recommendations. *Acad Med* 2014;89:1245–51.

30. Kon AA. The role of empirical research in bioethics. *Am J Bioeth* 2009;9:59–65.

31. Morberg Jämterud S. Human dignity: a study in medical ethics. PhD thesis. Uppsala University, Sweden, 2016.

32. Zeiler K, De Boer M. The empirical and the philosophical in empirical bioethics: time for a conceptual turn. *AJOB Empir Bioeth* 2020;11:11–3.

33. Ives J, Dunn M, Crabb A, eds. *Empirical bioethics: theoretical and practical perspectives*. Cambridge: Cambridge University Press, 2018.

34. Musschenga AW. Empirical ethics, context-sensitivity, and contextualism. *J Med Philos* 2005;30:467–90.

35. Mackenzie C, Stoljar N, eds. *Relational autonomy – feminist perspectives on autonomy, agency, and the social self*. New York: Oxford University Press, 2000.

36. Verkerk MA. The care perspective and autonomy. *Med Health Care Philos* 2001;4:289–94.

37. Dove ES, Kelly S, Lucivero F, et al. Beyond individualism: is there a place for relational autonomy in clinical practice and research? *Clin Ethics* 2017;12:150–65.

38. Sherwin S. A relational approach to autonomy in healthcare. In: Sherwin S, ed. *The politics of women's health: exploring agency and autonomy*. Philadelphia: Temple University Press 1998:19–47.

For peer review only - http://bmjopen.bmj.com/site/about/guidelines.xhtml

39. Zeiler K. Shared decision-making, gender and new technologies. *Med Health Care Philos* 2007;10:279–87.

40. Verkerk M, Lindemann H, McLaughlin J, eds. *What about the family?: practices of responsibility in care*. New York, NY: Oxford University Press, 2019.

41. Fitzgerald D, Callard F. Entangling the medical humanities. In: Whitehead A, Woods A, eds. *The Edinburgh companion to the critical medical humanities*. Edinburgh: Edinburgh University Press 2016:35–49.

42. Raz AE. *Community genetics and genetic alliances: eugenics, carrier testing, and networks of risk*. Abingdon, Oxon and New York: Routledge, 2010.

43. Raz AE, Amano Y, Timmermans S. Coming to terms with the imperfectly normal child: attitudes of Israeli parents of screen-positive infants regarding subsequent prenatal diagnosis. *J Community Genet* 2019;10:41–50.

44. De Wert GM, Dondorp WJ, Knoppers BM. Preconception care and genetic risk: ethical issues. *J Community Genet* 2012;3:221–28.

45. Briggs A, Nouri PK, Galloway M, et al. Expanded carrier screening: a current survey of physician utilization and attitudes. *J Assist Reprod Genet* 2018;35:1631–40.

For peer review only

Figure legends

Figure 1. The empirical bioethics discussion of the theme “Choice and its complexity” showed that several areas – medical, psychological, moral, social – co-shape the complexity of choice on PECS. All these areas need to be reflected on, not only by each individual but also by the couple, together. Hence the couple need to engage in a shared relational autonomous decision-making on these areas.

For peer review only

For peer review only - http://bmjopen.bmj.com/site/about/guidelines.xhtml

Figure 1. Result of analysis - Choice and its complexity and shared relational autonomous decision-making on PECS

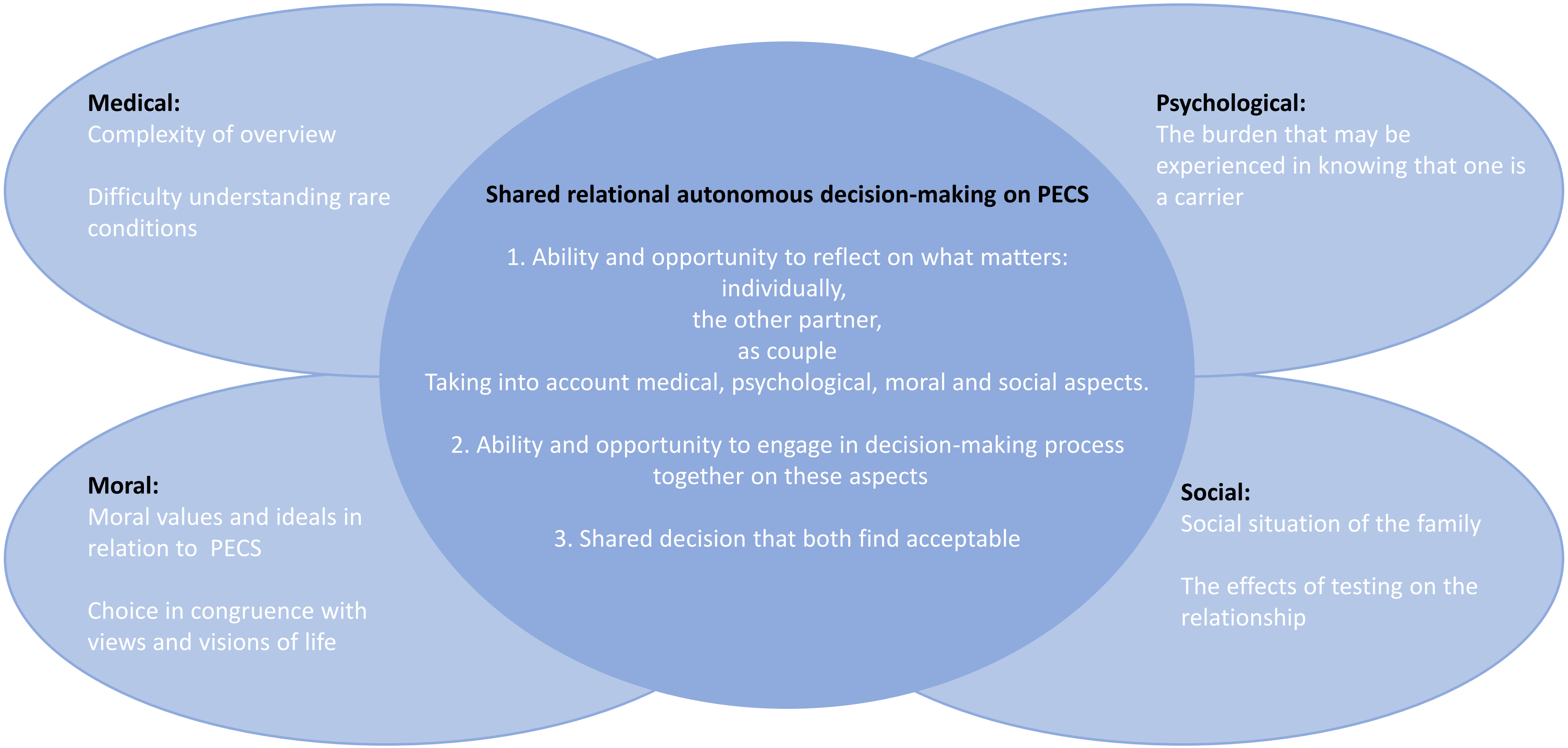

Supplementary file. Semi-structured interview-guide

(Title of article: *A qualitative study of GPs' views and experiences of population-based preconception expanded carrier screening in the Netherlands: Bioethical perspectives*)

Due to the semi-structured format of the interviews the topics and examples of questions are stated.

First impression of the test

1. Could you describe how you got involved in the PCS project? When did you first hear about it?

Implications of the test

2. Can you describe a general case when someone asks for the screening?

3. How do you explain to people what the test involves?

4. Could you describe different aspects of the test?

Experiences with patients

6. How did couples give meaning to a positive result?

7. How did couples give meaning to a negative result?

8. Did you discuss with the patients the different ways in which the results could be interpreted?

How the test could be improved

9. What do you think should be adjusted in how the test is offered now?

10. How do you think the future of the test looks like?

Other questions

Is there anything we haven't discussed that you would like to add?

For peer review only

Reporting checklist for qualitative study.

Based on the SRQR guidelines.

Instructions to authors

Complete this checklist by entering the page numbers from your manuscript where readers will find each of the items listed below.

Your article may not currently address all the items on the checklist. Please modify your text to include the missing information. If you are certain that an item does not apply, please write "n/a" and provide a short explanation.

Upload your completed checklist as an extra file when you submit to a journal.

In your methods section, say that you used the SRQRreporting guidelines, and cite them as:

O'Brien BC, Harris IB, Beckman TJ, Reed DA, Cook DA. Standards for reporting qualitative research: a synthesis of recommendations. Acad Med. 2014;89(9):1245-1251.

		Reporting Item	Page Number
**Title**			
	#1	Concise description of the nature and topic of the study identifying the study as qualitative or indicating the approach (e.g. ethnography, grounded theory) or data collection methods (e.g. interview, focus group) is recommended	1
**Abstract**			
	#2	Summary of the key elements of the study using the abstract format of the intended publication; typically includes background, purpose, methods, results and conclusions	2
**Introduction**			
Problem formulation	#3	Description and signifcance of the problem / phenomenon studied: review of relevant theory and empirical work; problem statement	4

For peer review only

Purpose or research question	#4	Purpose of the study and specific objectives or questions	5
**Methods**			
Qualitative approach and research paradigm	#5	Qualitative approach (e.g. ethnography, grounded theory, case study, phenomenolgy, narrative research) and guiding theory if appropriate; identifying the research paradigm (e.g. postpositivist, constructivist / interpretivist) is also recommended; rationale. The rationale should briefly discuss the justification for choosing that theory, approach, method or technique rather than other options available; the assumptions and limitations implicit in those choices and how those choices influence study conclusions and transferability. As appropriate the rationale for several items might be discussed together.	5-8
Researcher characteristics and reflexivity	#6	Researchers' characteristics that may influence the research, including personal attributes, qualifications / experience, relationship with participants, assumptions and / or presuppositions; potential or actual interaction between researchers' characteristics and the research questions, approach, methods, results and / or transferability	5,6,18
Context	#7	Setting / site and salient contextual factors; rationale	5
Sampling strategy	#8	How and why research participants, documents, or events were selected; criteria for deciding when no further sampling was necessary (e.g. sampling saturation); rationale	5,6,16
Ethical issues pertaining to human subjects	#9	Documentation of approval by an appropriate ethics review board and participant consent, or explanation for lack thereof; other confidentiality and data security issues	6-7

For peer review only

Data collection methods	#10	Types of data collected; details of data collection procedures including (as appropriate) start and stop dates of data collection and analysis, iterative process, triangulation of sources / methods, and modification of procedures in response to evolving study findings; rationale	5-8
Data collection instruments and technologies	#11	Description of instruments (e.g. interview guides, questionnaires) and devices (e.g. audio recorders) used for data collection; if / how the instruments(s) changed over the course of the study	5,6
Units of study	#12	Number and relevant characteristics of participants, documents, or events included in the study; level of participation (could be reported in results)	5,6
Data processing	#13	Methods for processing data prior to and during analysis, including transcription, data entry, data management and security, verification of data integrity, data coding, and anonymisation / deidentification of excerpts	5,6
Data analysis	#14	Process by which inferences, themes, etc. were identified and developed, including the researchers involved in data analysis; usually references a specific paradigm or approach; rationale	6
Techniques to enhance trustworthiness	#15	Techniques to enhance trustworthiness and credibility of data analysis (e.g. member checking, audit trail, triangulation); rationale	6-8
**Results/findings**			
Syntheses and interpretation	#16	Main findings (e.g. interpretations, inferences, and themes); might include development of a theory or model, or integration with prior research or theory	8-15

For peer review only

Links to empirical data	#17	Evidence (e.g. quotes, field notes, text excerpts, photographs) to substantiate analytic findings	8-15
**Discussion**			
Intergration with prior work, implications, transferability and contribution(s) to the field	#18	Short summary of main findings; explanation of how findings and conclusions connect to, support, elaborate on, or challenge conclusions of earlier scholarship; discussion of scope of application / generalizability; identification of unique contributions(s) to scholarship in a discipline or field	15-17 (in empirical bioethics this discussion is also included in the result
Limitations	#19	Trustworthiness and limitations of findings	16
**Other**			
Conflicts of interest	#20	Potential sources of influence of perceived influence on study conduct and conclusions; how these were managed	18
Funding	#21	Sources of funding and other support; role of funders in data collection, interpretation and reporting	18

The SRQR checklist is distributed with permission of Wolters Kluwer © 2014 by the Association of American Medical Colleges. This checklist was completed on 01. July 2020 using https://www.goodreports.org/, a tool made by the EQUATOR Network in collaboration with Penelope.ai

For peer review only
